# Malignant Pelvic Pheochromocytoma Presenting as NonFunctioning Kidney and Accelerated Hypertension: A Rare Presentation

**DOI:** 10.1155/2014/985615

**Published:** 2014-06-16

**Authors:** Santosh Kumar, Kalpesh Mahesh Parmar, Seema Prasad, Jyotsna Rani

**Affiliations:** ^1^Department of Urology, Postgraduate Institute of Medical Education and Research, Sector 12, Chandigarh 160012, India; ^2^Anaesthesia, Chandigarh, India

## Abstract

Paragangliomas are neuroendocrine tumors that arise from sympathetic nerve ganglia. They can develop anywhere from the neck to the pelvis, but are most commonly found in the abdomen, particularly at the aortic bifurcation or in the periaortic region. Malignant paragangliomas account for 29–40% of cases. We report a case of 36-year hypertensive female presented with and right flank pain and accelerated hypertension. On evaluation she was diagnosed to have non unctioning kidney due to malignant pelvic paraganglioma with right ureteric encasement. We believe our case is one of the first reported in literature as rare presentation of malignant paraganglioma presenting as nonfunctioning kidney and accelerated hypertension.

## 1. Introduction

Paragangliomas are malignant tumors that arise from the extra-adrenal paraganglionic cells of the sympathetic or parasympathetic systems. They can be observed from the base of the skull to the bladder or along the sympathetic ganglionar chain. Retroperitoneal paragangliomas can represent a truesurgical challenge due to their tight relation to largesize vessels. We present a case of malignant pelvic mass presenting as nonfunctioning kidney and accelerated hypertension. Its final histologic diagnosis was paraganglioma that was functional with metastasis to paraaortic nodes.

## 2. Case Report

**Figure 1:**
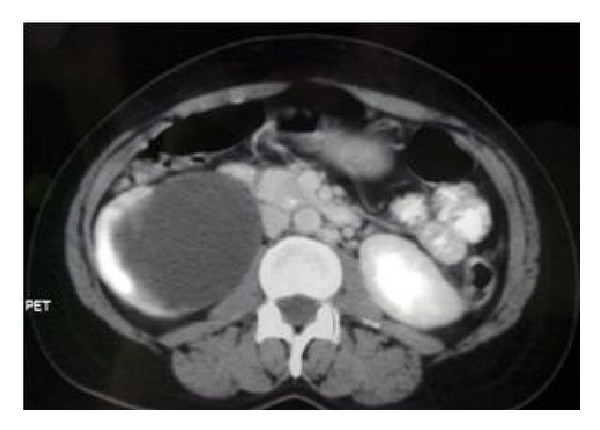
PET CT axial cut showing gross hydronephrosis of right kidney with thinned out parenchyma and left mild hydronephrosis.

**Figure 2:**
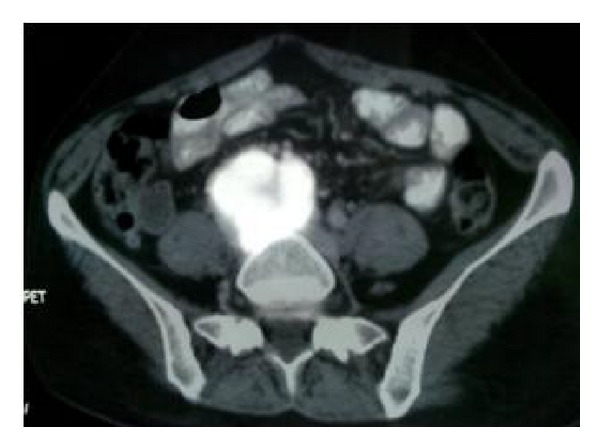
PET CT axial cut showing intense tracer uptake in the pelvic mass abutting the right psoas muscle.

**Figure 3:**
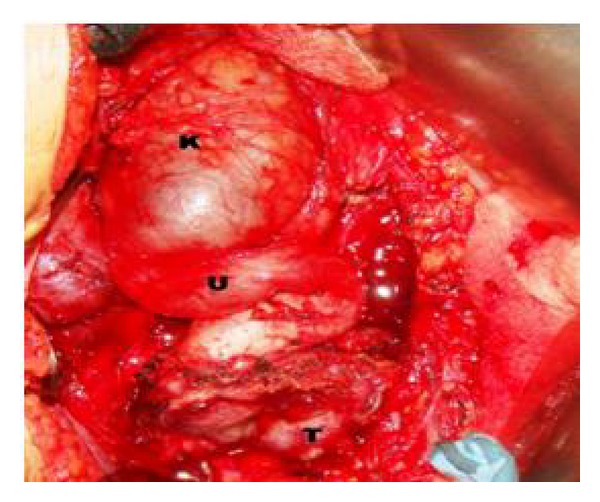
Intraoperative image showing gross hydroureteronephrosis with tortuous ureter and tumor with ureteric encasement.

**Figure 4:**
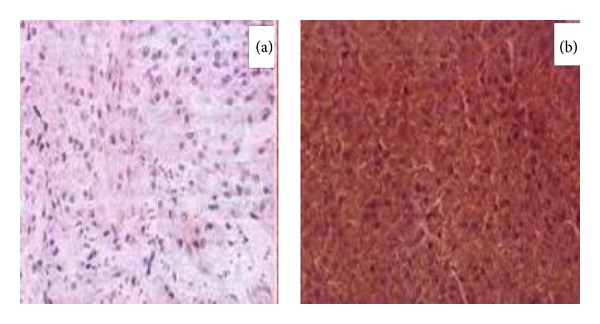
Immunochemistry results showing presence of S-100 (a) and chromogranin markers (b).

A 36-year-old hypertensive female on multiple antihypertensive medications presented to our institute with complaints of right flank pain of 2-month duration. No history of paroxysmal hypertension, sweating, headaches, hematuria, dysuria, fever, and bowel complaints was present. There was no significant family history and no history of sudden unexplained death in the family. On general physical examination, pulse was 98/min and BP was 174/90 mmHg. On per abdomen examination no mass was palpable. Serum biochemistry showed serum creatinine of 2.1. Rest of the routine work-up was normal. USG abdomen showed gross hydronephrosis of right kidney with suspected pelviureteric junction obstruction. Renal dynamic scan revealed nonfunctioning right kidney. Patient was admitted with plan for right nephrectomy as cause of accelerated hypertension. However, her blood pressure was uncontrolled. On further evaluation her serum metanephrines levels were found to be 660 pg/mL (normal range 0–58 pg/mL). Serum normetanephrines levels were normal. CECT abdomen revealed heterogeneously hypodense mass lesion 7 × 6 × 4 cm at the level of L4-5 level with areas of central necrosis and peripheral rim enhancement abutting bilateral common iliac vessels near bifurcation; ureter and adjacent vertebral body multiple enlarged lymph nodes were present in paraaortic and paracaval region. Right kidney was grossly hydronephrotic with thinned out cortex and left kidney showed mild hydronephrosis (Figure 1). 68 Ga DOTATATE PET CT showed intense tracer uptake and somatostatin receptor expressing soft tissue mass extending from the level of the aortic bifurcation to the lower border of the L5 vertebra (Figure 2). Paraaortic nodes also showed intense tracer uptake suggestive of metastasis. Patient was started on tablet phenoxybenzamine 10 mg twice a day, two weeks prior to surgery, and subsequently atenolol 10 mg once a day was added. Patient was optimized and taken up for surgery. Adequate precautionary measures were taken by the anaesthetist team in perioperative period. Patient underwent exploratory laparotomy via midline incision. Mass was localized at the level of aortic bifurcation and densely adherent to common iliac vessels bilaterally, and right ureter was completely encased by the mass. Multiple paraaortic enlarged lymph nodes compressing left ureter were present. Careful dissection and excision of the mass were done preserving the major vessels and left ureter. Right nephroureterectomy was done en bloc with pelvic mass (Figure 3). Paraaortic lymph node was removed as well. Grossly the major bulk of tumor and lymph nodes were removed. Postoperative period was uneventful. Histopathology report revealed grossly the tumor mass with right nephroureterectomy specimen. Margins were negative for tumor cells. Tumor cells were arranged in nest, separated by vascular septae, round to polygonal, with amphophilic eosinophilic cytoplasm suggestive of paraganglioma. There was nuclear hyperchromasia, moderate nuclear pleomorphism, and high mitotic count with atypical mitotic figures suggestive of malignancy. Immunochemistry results showed S-100 and chromogranin markers positive (Figure 4). Paraaortic nodes revealed metastatic tumor deposit.

## 3. Discussion

Pheochromocytomas are tumors that arise from chromaffin cells of the adrenal medulla. They are called paraganglioma if chromaffin-cell tumors originate from extra-adrenal sites along the sympathetic and/or the parasympathetic chain [1]. Paragangliomas may be located at any place from the cervical region to the pelvic cavity [2]. They may present at all ages with a peak incidence around 30–50 years. Extra-adrenal paraganglioma rate of malignancy is more than intra-adrenal paragangliomas, especially if related to succinate dehydrogenase B mutations (SDHB) [3, 4]. One study showed that 55% of paragangliomas are malignant, and 83.3% of them had genetic mutation [5]. This is in contrast to the intra-adrenal paraganglioma whose malignancy rate is 10% [6]. Diagnosis of pheochromocytoma/paraganglioma is usually performed by clinical presentation and elevated catecholamine levels of serum and/or urine or their metabolites [6]. High plasma levels of chromogranin A have been suggested to be indicative of malignancy [7]. Although paragangliomas are catecholamine secreting tumors, 17% of cases are asymptomatic [8]. In the state of secretory tumors the most common symptoms are headache, sweating, tachycardia or palpitations, dyspnea, nausea, and chest pain [6]. The asymptomatic cases pose a diagnostic dilemma. As was our case, patient presented with right dull aching flank pain and was initially diagnosed to have right gross hydronephrosis and renal dynamic scan documented nonfunctioning right kidney. Patient was planned for right nephrectomy as a cause for causal hypertension. However in patients with uncontrolled hypertension, further work-up is essential to rule out systemic causes. In our case, patient's serum metanephrines level was raised and CECT abdomen was done which revealed a pelvic mass with enlarged paraaortic lymph nodes. With the suspicion of functional paraganglioma, patient was started on *α* and *β* blocker. Surgical excision of the tumor with involved tissues is the primary treatment of choice because these tumors are commonly chemo- and radioresistant [6]. Patient was taken up for exploratory laparotomy and en-bloc excision of mass with right nephroureterectomy was done (Figure 3). The only absolute indication of malignancy may be distant metastasis to the organs such as liver, bone, and lymph nodes. Certain histological features such as invasion; vascular and/or capsular, confluent or focal necrosis, diffuse growth or large nests, high cellularity nuclear pleomorphism, and hyperchromasia are suggestive of malignancy [2]. Several possible characteristics are suggested as predictors of malignancy: tumor weight more than 80 g, high concentration of dopamine inside the tumor, and tumor size more than 5 cm (75% predictive) [9]. All paragangliomas should be regarded as potentially malignant because of difficulty in confirming malignancy. Patients with malignant paragangliomas are cured only by surgery [2].

Patient who present with nonfunctioning kidney and accelerated hypertension should be worked up to rule out other systemic causes which may be missed if work-up is not done. As was our case, patient was uncontrolled on four antihypertensive medications, and on work-up she was found to have pelvic paraganglioma. Histopathology revealed malignant paraganglioma with metastasis in paraaortic lymph nodes. Patient was followed up with 68 Ga DOTATATE PET CT at three months which showed no somatostatin receptor uptake in the pelvis and rest of the body, suggesting no recurrence or metastasis. Patient is started on adjuvant chemotherapy cyclophosphamide, vincristine, and dacarbazine. Genetic testing is being planned for the patient as well as family members in the follow-up period.
